# Current Risk of Dirofilariosis Transmission in the Iberian Peninsula (Spain and Portugal) and the Balearic Islands (Spain) and Its Future Projection under Climate Change Scenarios

**DOI:** 10.3390/ani13111764

**Published:** 2023-05-26

**Authors:** Iván Rodríguez-Escolar, Ricardo E. Hernández-Lambraño, José Ángel Sánchez-Agudo, Manuel Collado, Patricia Pérez-Pérez, Rodrigo Morchón

**Affiliations:** 1 Zoonotic Diseases and One Health GIR, Biomedical Research Institute of Salamanca-Research Centre for Tropical Diseases University of Salamanca (IBSAL-CIETUS), Faculty of Pharmacy, Campus Miguel Unamuno, University of Salamanca, 37007 Salamanca, Spain; ivanrodriguez@usal.es (I.R.-E.); manuelcollado@usal.es (M.C.); patriciaperez2510@gmail.com (P.P.-P.); 2 Biodiversity, Human Diversity and Conservation Biology Research Group, Campus Miguel Unamuno, University of Salamanca, 37007 Salamanca, Spain; ricardohl123@usal.es (R.E.H.-L.); jasagudo@usal.es (J.Á.S.-A.)

**Keywords:** ecological niche model, forward projection, *Dirofilaria* spp., *Culex pipiens*, Spain, Portugal, Iberian Peninsula

## Abstract

**Simple Summary:**

Dirofilariosis (*Dirofilaria* spp.) is a vector-borne zoonotic disease that mainly affects canids and felids, both domestic and wild, and accidentally humans. In Spain and Portugal, it is considered an endemic disease where the prevalence is not uniform throughout the territory. The objective is to carry out a quantitative proposal of the risk of infection by *Dirofilaria* spp., using as key variables the potential distribution of suitable habitats for *Culex pipiens* calculated via an ecological niche model (ENM) and the potential number of *Dirofilaria* spp. generations. In addition, the impact of possible future climatic conditions was estimated to the periods of the 2040s, 2060s, and 2080s. The resulting model was validated with the prevalence and geolocation of *D. immitis*-infected dogs from all provinces and districts. The risk of *Dirofilaria* spp. infection was high throughout the peninsula and the Balearic Islands, with the exception of higher-altitude areas. We found a robust and positive relationship between the risk of dirofilariosis and observed the prevalence of infested dogs in the study area. The territory gain of *Cx. pipiens* will increase by 49.98%, potentially increasing the risk. This new model increases the accuracy and predictive value of existing models.

**Abstract:**

Dirofilariosis is a vector-borne zoonotic disease whose distribution is linked to the presence of culicid mosquitoes. Spain and Portugal are considered endemic countries; however, the distribution of dirofilariosis is not uniform. Our aim was to develop a more accurate risk model of dirofilariosis transmission for the Iberian Peninsula (Spain and Portugal) and the Balearic Islands (Spain). To do this, we used a set of key variables related to parasite transmission: the potential distribution of suitable habitats for *Culex pipiens* calculated via an ecological niche model (ENM) and the potential number of *Dirofilaria* spp. generations. The resulting model was validated with the prevalence and geolocation of *D. immitis*-infected dogs from all provinces and districts. In addition, the impact of possible future climatic conditions was estimated. A quantitative estimate of the risk of infection by *Dirofilaria* spp. was obtained at a resolution of 1 km^2^. The entire analyzed territory was susceptible to contact with the parasite. The highest risk of infection was found throughout the eastern coastal strip and the south of the Iberian Peninsula and the Balearic Islands, as well as in the areas surrounding the basins of the main rivers, and the lowest risk was located in the higher-altitude areas. We found a robust and positive relationship between the risk of dirofilariosis and the observed prevalence of infested dogs in the study area (β ± SE = 3.32 ± 1.43 *p* < 0.05). In 2080, the percentage of territory gain for *Cx. pipiens* will increase to 49.98%, which will increase the risk of infection. This new model provides a high predictive value for the current and predicted presence and risk and can serve as a tool for the management and control of dirofilariosis.

## 1. Introduction

Anthropogenic global warming is causing disturbances in terrestrial and inland freshwater aquatic ecosystems and coastal zones in all climatic regions of Europe [1]. One of the consequences of these disturbances is the increased risk of transmission of vector-borne diseases affecting both animal and human populations [2]. Changes in water and temperature regimes directly influence the development of vectors and may increase their distribution range and lengthen the annual periods during which they are active [1].

Among these diseases is dirofilariosis, a vector-borne zoonotic disease caused by different species of the genus *Dirofilaria* spp. of which *D. immitis* and *D. repens* are the most important [3]. Canids and felids, both domestic and wild, act as definitive hosts [4,5] with culicid mosquitoes of the genera *Culex* spp., *Aedes* spp., and *Anopheles* spp. as vectors [5,6]. In addition, humans can also be infected, in the same areas where microfilaremic reservoirs exist, resulting in human dirofilariosis [3].

It is a cosmopolitan disease, mainly localized in tropical and semi-tropical regions worldwide, areas particularly sensitive to climatic changes, where changes in its distribution pattern are being observed [3,4,5,7,8]. In Europe, its presence is steadily expanding, and it is now endemic in southern and north-central countries [5]. Its distribution is directly linked to the presence of vectors, whose life cycle is in turn closely associated with the existence of freshwater bodies (rivers, irrigation, and stagnant water areas) and climatic factors (humidity and temperature), as the molting period of larvae in the vector is shortened when the ambient temperature increases [4,5,9,10,11,12].

Spain and Portugal have traditionally been considered endemic countries, although not all areas of the Iberian Peninsula have been studied [5]. In Spain, the prevalence of *D. immitis* in dogs is 6.47% [13], and the seroprevalence in cats is 9.4% [14]. In the case of Portugal, published studies in dogs provide prevalence values ranging from 0.9% to 27.3%, being higher in the south [15,16,17,18,19,20]. However, *D. repens* infections in the Iberian Peninsula have not been widely reported, where very few cases of microfilaremic dogs have been reported in Spain and one in Portugal [21,22,23,24]. In relation to humans, very few cases of individuals infected by *Dirofilaria* spp. have been reported. In fact, only a few cases have been reported in Spain, most of them originating from *D. repens* (subcutaneous nodules) and only a few cases from *D. immitis* (pulmonary nodule) [3,8,25,26,27]. Finally, as regards the study of vectors, there are only studies in which *Cx. pipiens* has been described as a vector transmitting the disease in the Iberian Peninsula [28,29,30].

Among the measures that can be considered to control this disease, as with other diseases that are also mediated by vectors of transmission, is the prediction of the dispersal capacity of these agents through ecological niche modeling (ENM) processes. As a biological species, they are assumed to respond to environmental factors, of which bioclimatic characteristics are the most important [31,32]. Determining where the most favorable circumstances for their existence are found can be a major advance in the best ability to deal with them preventively. ENMs are ecoinformatics-type tools that assign suitability values in the environmental space of a species’ distribution, according to the correlation calculated between the presence/absence of the modeled event, in this case, the known distribution records of the species, and the environmental variables to which it responds [33]. Translating this environmental envelope to the geographic space corresponding to the region under analysis results in a potential distribution map of habitat suitability [34,35,36,37,38,39]. Among the many modeling approaches, one of the most established is the maximum entropy algorithm (Maxent), which works with occurrence data and builds very statistically robust models, even with few occurrence points of the species studied [32,37,40,41,42].

In this methodological field of ENMs, we are hardly aware of any studies in relation to parasitic diseases and their vectors [43,44,45,46,47,48,49,50,51,52,53,54,55,56] and none in relation to dirofilariosis. Most of the studies that analyze the risk of infection by *Dirofilaria* spp. that incorporate cartographic information, on one hand, are restricted to the European region and, on the other hand, only deal with spatial interpretation using Geographic Information Systems (GIS) based mainly on temperature records [7,10,55,56,57,58,59,60,61,62,63]. In Spain, there is a national study offering a map of the potential risk of infection [7] and another for the periurban territory of Barcelona [64], both using a simple geoenvironmental model based solely on temperature, rainfall, and the distribution of irrigated crops, where the highest risk values are related to the high presence of irrigated areas and/or coastal areas with high humidity. It is known that among the most important factors determining the transmission of the disease are the optimal environmental conditions for the development of insect vectors. Insects are heterothermic organisms that are highly dependent on environmental variables to activate their metabolism and behavior. An extensive literature on flying insects reports the simultaneous effects of temperature and many other weather variables, such as precipitation or wind speed, on the breeding, abundance, survival, and activity of hematophagous species. In this respect, incorporating a variable that describes the distribution patterns of its main vector into risk models will offer significant improvements in model accuracy.

Taking into account the health relevance of these zoonotic diseases and their apparent current expansion process, largely favored by anthropogenic alterations (such as the aforementioned global warming), it is necessary to develop detailed analyses that focus on the environmental circumstances that promote them. The dimension of spatially explicit risk models allows us to establish correlations between the presence of zoonotic diseases and the variables associated with their transmission, correlations that can be extrapolated to other territories where data do not exist and also to other time frames, in order to be able to anticipate their future dynamics in time and take precautionary measures to tackle them before they occur. Thus, the objective of this study was to develop a more accurate risk model of dirofiariosis transmission in the Iberian Peninsula (Spain and Portugal) and the Balearic Islands (Spain). For this purpose, we used a set of key variables associated with parasite transmission: the potential distribution of suitable habitats for *Cx. pipiens*, calculated with ENMs, and the potential number of *Dirofilaria* spp. generations. The incorporating of suitable habitat for *Cx. pipiens* as a critically important variable in the risk model represents a novel contribution to substantially improve the predictive power of existing studies.

## 2. Materials and Methods

### 2.1. Iberian Peninsula (Spain and Portugal) and the Balearic Islands (Spain)

**Figure 1:**
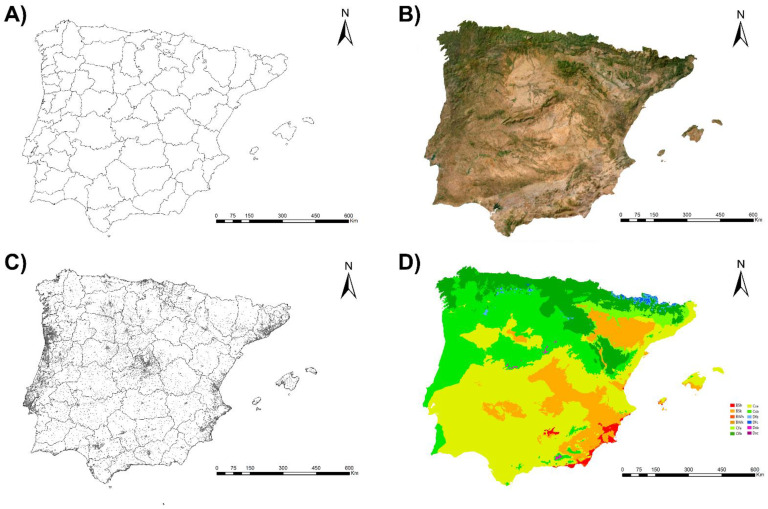
Spain and Portugal and Balearic Islands (Spain) according to their (**A**) provinces and districts, (**B**) orography, (**C**) location of human populations, and (**D**) climates according to the Köppen Climate Classification System (BSh: hot semi-arid climate; BSk: cold semi-arid climate; BWh: hot desert climate; BWk: cold desert climate; Cfa: humid subtropical climate; Cfb: temperate oceanic climate; Csa: hot-summer Mediterranean climate; Csb: warm-summer Mediterranean climate; Dfb: humid continental climate; Dfc: subarctic climate; Dsb: humid continental climate; Dsc: subarctic climate).

The area considered for this study was the Iberian Peninsula (40°14′24″ N 4°14′21″ W) and the Balearic Islands. It is a territory that constitutes the southeastern tip of Europe, separated by the Strait of Gibraltar from North Africa (Figure 1). Although several islands, such as Madeira, the Azores, and the Canary Islands, correspond politically to Spain and Portugal, due to their clearly different biogeographical situation, they have not been taken into account in this work. The Iberian Peninsula is surrounded to the east and south by the Mediterranean Sea, to the north by the Cantabrian Sea, and to the west and south by the Atlantic Ocean. It covers an area of approximately 590,000 km^2^, with the Balearic Islands covering 4992 km^2^. Most of the territory is defined by the presence of a high plateau with an average altitude of 600 m, slightly tilted to the west. It is crossed by several large rivers, whose basins are well marked, such as the Guadalquivir and Ebro river basins (northeast) and other shallower basins such as the Miño (northwest), Duero and Tajo (west), Guadiana (southwest), and Júcar and Seguro (east). The main mountain systems are the Sistema Central, Sistema Ibérico, Cordillera Cantábrica, Pyrenees, Sistema Ibérico, and Sistema Penibético.

The Iberian Peninsula has a wide variety of climates throughout its territory due to its location between Europe and Africa and its orographic diversity. In general, the Mediterranean bioclimate predominates, with only the northern fringe being clearly Euro-Siberian in character. Inland, the climate is markedly continental with low winter temperatures, high summer temperatures, and long periods of summer drought. The Atlantic strip, however, has an oceanic climate with more moderate temperatures and abundant rainfall [65].

### 2.2. Cx. pipiens Habitat Suitability Modelling

#### 2.2.1. *Cx. pipiens* Data Collection

We used occurrence points of *Cx. pipiens* from all Iberian Peninsula provinces (Spain and Portugal) from data obtained by Gangoso et al. [2] for Spain and by Ferreira et al. [29] together with distributional records for the species from GBIF [66] for Portugal due to the few records of the species from this country. The presence data were processed in 1 km^2^ spatial resolution to avoid biases in vector presence data collection. This compiled database was the result of an extensive search of the occurrence of *Cx. pipiens* in the study area. The spatial independence of these datasets was an important underlying assumption of this study as it allowed an accurate representation of the observed occurrence of the species in the study area. However, we acknowledge that this database may not represent the full range of climatic conditions in which the species can be found outside of the study area. Thus, our estimates will always be a conservative representation of the full potential distribution of the species.

#### 2.2.2. Environmental and Bioclimatic Data

The bioclimatic data, including 19 bioclimatic variables related to temperature and precipitation, were downloaded from the World Clim [67] in 1 km^2^ spatial resolution for the current data (1970–2000) as well as the predicted data for the 2040s, 2060s, and 2080s [1]. To avoid cross-correlation between the 19 selected bioclimatic variables, a multicollinearity test was carried out in R software using Pearson’s correlation coefficient [68]. Variables with cross-correlation coefficient values of r > ±0.75 were excluded. The bioclimatic variables selected, according to vector biology, were the annual mean temperature (°C) (BIO_1_), isothermality (BIO_3_), temperature seasonality (*SD* × 100) (BIO_4_), mean temperature of the wettest quarter (°C) (BIO_8_), mean temperature of the driest quarter (°C) (BIO_9_), annual precipitation (mm) (BIO_12_), and precipitation seasonality (coefficient of variation) (BIO_15_).

The human footprint [69], the distribution of irrigated crop areas, the locations of water bodies and rivers [70], and the herbaceous plant and shrub density [71] were used due to their effect on *Cx. pipiens* distribution. In terms of the human footprint, it represented a global map of cumulative human pressure on the environment. Human pressure was measured using eight variables including built environment, population density, electric power infrastructure, cropland, grazing land, roads, railways, and waterways.

#### 2.2.3. Modeling Approaches

We used the Maxent program [72] to model the habitat suitability and potential geographical distribution of *Cx. pipiens* within the study area. This algorithm based on the maximum entropy principle calculated the habitat suitability of a species as a function of environmental constraints [73]. For choosing an appropriate amount of model complexity, we used the KUENM package in R [74], which selected the best Maxent models of a series of candidates arranged by different combinations of parameter settings. For our study, we generated 119 models using a set of climate variables, 17 values of the regularization multiplier (0.1–1.0 at intervals of 0.1, 2–6 at intervals of 1, and 8 and 10), and the seven possible combinations of three feature classes (linear, quadratic, and product). The model performance was assessed in terms of statistical significance (Partial_ROC < 0.05), omission rates (OR = 5%), and model complexity using the Akaike information criterion corrected for small sample sizes (AICc). Significant models with an omission rate ≤ 5% were selected. Then, from this set of models, those with an AICc delta value of ≤2 were selected as the final candidate models. The candidate models were built using the “kuenm_cal” function, and the evaluation and selection of the best model were carried out using the “kuenm_ceval” function.

We generated the final ENM (best-fit model) using the variables and the same parameters as previously selected. Ten bootstrap replications with logistic outputs were performed. The evaluation of these final models was based on the ROC_partial, OR, and AICc calculations using an independent dataset. The creation of the final models was carried out by using the “Kuenm_mod” function.

### 2.3. Dirofilaria Spp. Generations

The annual generations of *Dirofilaria* were used as a weighting variable in the risk model. They were calculated using the model described by Simón et al. [7] and Genchi et al. [55] in R-4.3.0 software. This model estimated that the full development of *Dirofilaria* L3 in mosquito vectors (extrinsic incubation) needed the accumulation of 130 growing degree days (GDDs), in a period of 30 days, the maximum expectancy life of the vector species. Each day accumulated a number of GDDs equivalent to the degrees by which the mean daily temperature exceeded 14 °C. The threshold of 130 GDDs was accepted only if it was reached in 30 consecutive days. The mean daily temperature data [75] from 1990 to 2016 were used to calculate the number of generations of *Dirofilaria* spp. in the different territories of the Iberian Peninsula and Balearic Islands [76] from the R software.

### 2.4. Dirofilaria Spp. Risk Map and Its Validation

To create a risk map of *Dirofilaria* spp., we multiplied (weighting approach) the final *Cx. pipiens* ENM and the *Dirofilaria* spp. generations using the raster calculator in ArcMap 10.8.

For the validation of the *Dirofilaria* spp. risk map, we performed a regression calculation in R software between the mean risk of infection and disease prevalence in dogs most recently in all provinces of the Iberian Peninsula and in the Balearic Islands (Spain) [13] and all Portuguese districts of the Iberian Peninsula, except Leira district where no data existed [77]. In addition, dogs infected with *D. immitis* were geolocated.

### 2.5. Forward Projection and Rank Change Analysis

To assess the potential impact of global warming on the dynamics of Dirofilaria transmission risk, we forecasted the best ENM of *Cx. pipiens* by using the projection of the climate variables analyzed for three periods: the 2040s (2021 to 2040) the 2060s (2041 to 2060) and the 2080s (2061 to 2080), the representative concentration pathway (RCP) 8.5, which represents high CO_2_ emissions, and one global circulation models [78]. This model is among the most-used models currently available for simulating the global climate response to increasing greenhouse gas concentration in European continent [79].

Once the projections were generated, to determine changes in suitable habitats for *Cx. pipiens* in the Iberian Peninsula and the Balearic Islands, we converted the ENM and future projections into binary presence/absence maps using the equal training sensitivity and specificity cumulative threshold. This threshold maximized the agreement between the observed distribution of species and the predicted distribution calculated by the model. A range shift analysis was carried out using biomod2 in the R program, to establish those territories where a change in the distribution of *Cx. pipiens* will occur as a consequence of climate change [80]. This analysis consisted of calculating the percentage of cells that gained or lost climatic suitability for the models projected to 2024, 2060, and 2080, compared with the current area.

## 3. Results

### 3.1. Habitat Suitability Model for Cx. pipiens

**Figure 2:**
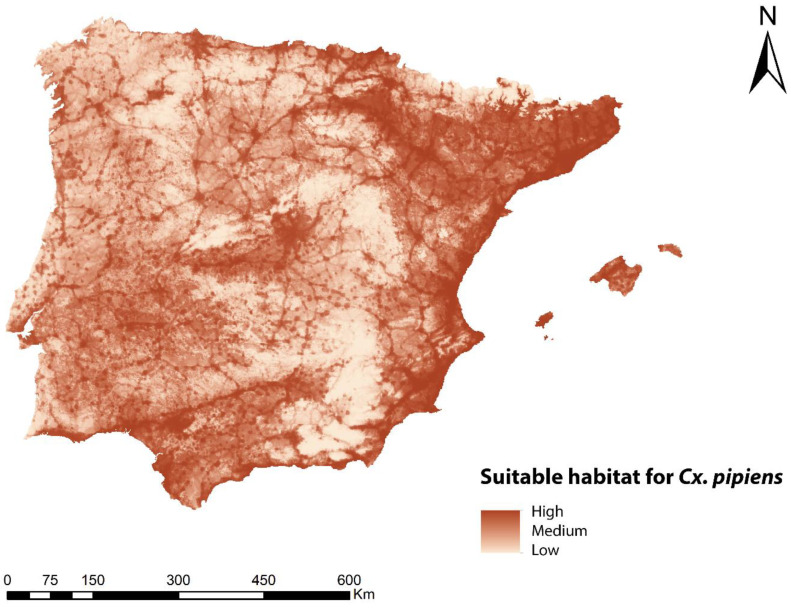
Ecological niche model for *Cx. pipens* in the geographical area of the Iberian Peninsula and Balearic Islands representing suitable habitat.

An ENM was developed for *Cx. pipens* in the geographical area of study. Figure 2 shows the map with the habitat suitability for this species, with a maximum value of 0.89 (high suitability) and a minimum value of 0.0022 (low suitability). The contribution of each bioclimatic and environmental variable to the ENM for *Cx. pipens* is shown in Table 1, and its visual representation is shown in Supplementary Figures S1–S13. The variables with the highest percentage contribution were the human footprint and BIO8, being 56.7% and 27.2%, respectively. The rest of the variables had values lower than 3.6%. According to the map, the areas with the best habitat suitability for *Cx. pipens* were mainly located in the northwest of the peninsula, in areas close to the Mediterranean Sea (east and south), and in the center of the peninsula, as well as in locations with a high intensity of anthropic use. On the contrary, mountainous areas, due to being less populated and less cultivated, as well as a large part of the east and northwest of the Iberian Peninsula, showed a low capacity to host *Cx. pipens*.

### 3.2. Number of Generations of Dirofilaria Spp.

**Figure 3:**
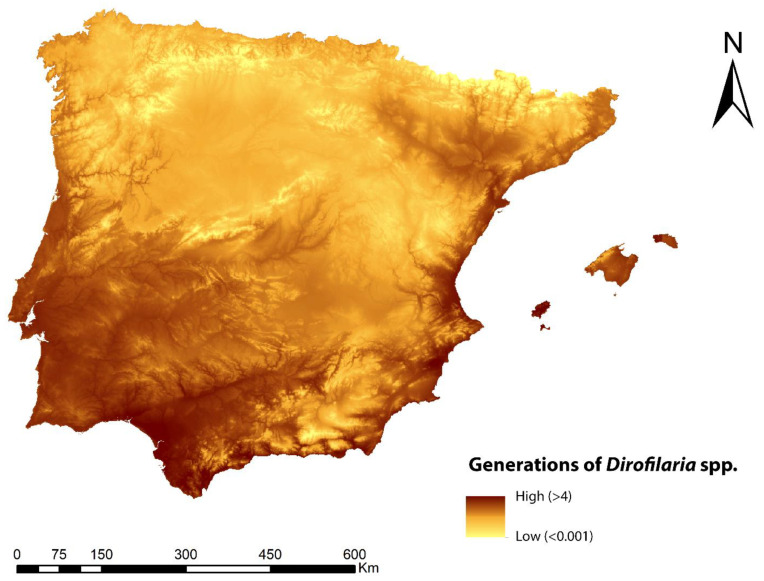
Prediction of the number of generations of *Dirofilaria* spp. in the Iberian Peninsula and Balearic Islands.

The predicted spatial distribution of the number of generations of *Dirofilaria* spp. is shown in Figure 3. The highest number of generations (greater than four) was found in the coastal areas of the Mediterranean Sea, Ibiza (Balearic Islands), and practically all of the southeast of the Iberian Peninsula. Inland areas showed values mostly between two and three generations, as did the northwest and north coast of the peninsula. In general, the mountainous zones and surrounding areas showed low values of between 1 and 0.1.

### 3.3. Potential Risk of Transmission of Dirofilaria spp.

**Figure 4:**
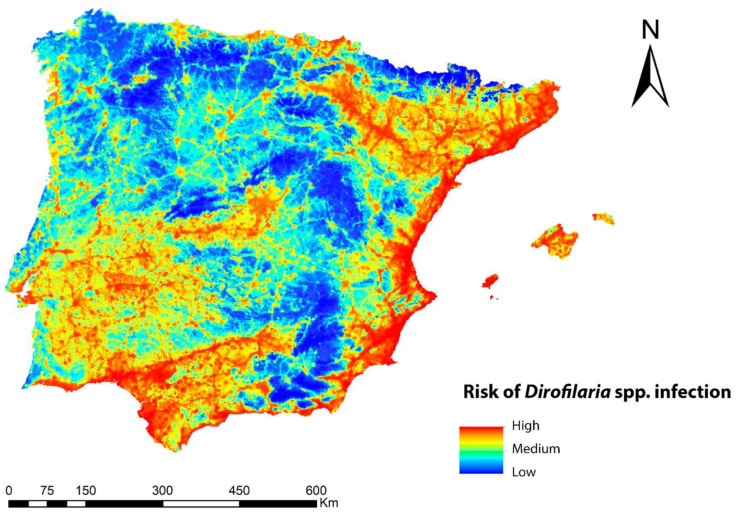
Ecological niche model of the risk of *Dirofilaria* spp. infection in the Iberian Peninsula and the Balearic Islands.

The infection risk map for *Dirofilaria* spp. in the geographical area of the Iberian Peninsula and Balearic Islands is shown in Figure 4 and reflects, also in a range of colors, the different values of the potential risk of transmission of the zoonosis. In general, the risk was high throughout the Iberian Peninsula and the Balearic Islands, with the exception of the higher-altitude areas. Five ranges of values were established, according to which 2.5% of the territory was in the upper range, implying a high risk of transmission; 10% was in the second range, implying a medium-high risk; 17.3% was in the third range with a medium-low risk; and 35.5% was in the fourth range and indicated a low risk, with 36.7% being very low. Regions with high risk values were abundant throughout the Mediterranean strip and the south, as well as in inland areas of the southwest, center, and north of the peninsula where the number of generations of *Dirofilaria* spp. and suitable habitats for *Cx. pipiens* were high. It was also high in all the basins of the main peninsular rivers. Medium-prevalence risks occurred in locations with a smaller human footprint, low number of generations of *Dirofilaria* spp., and low suitable habitats for *Cx. pipiens*, a factor that may help explain why mountainous areas had the lowest risk of infection.

### 3.4. Validation of the Dirofilaria spp. Transmission Risk Model

**Figure 5:**
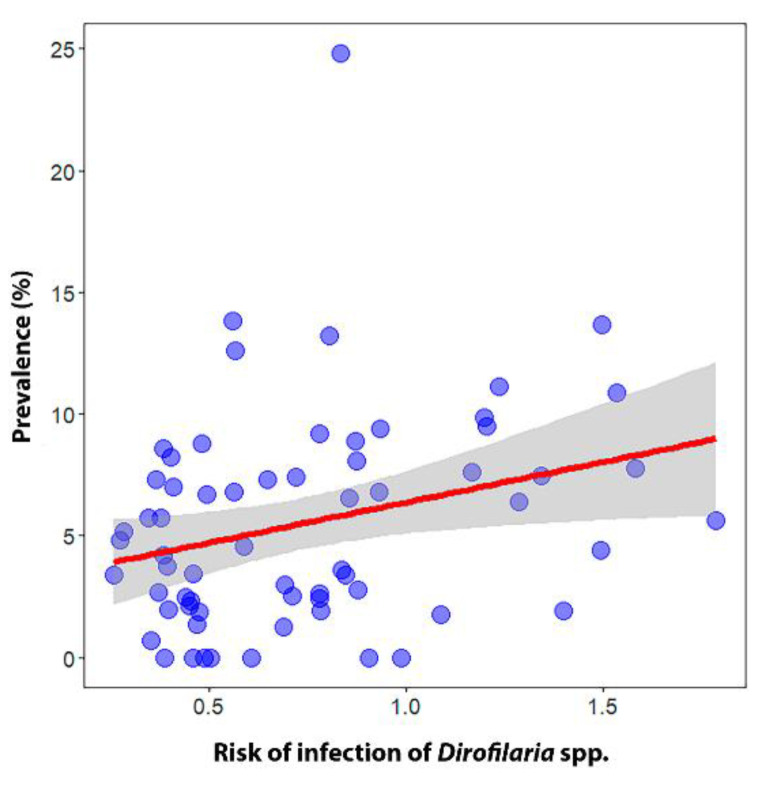
Regression plot for the validation of the ecological niche model between the mean risk of infection and disease prevalence in dogs most recently to date in all Spanish provinces and Portuguese districts of the Iberian Peninsula and in the Balearic Islands (Spain).

**Figure 6:**
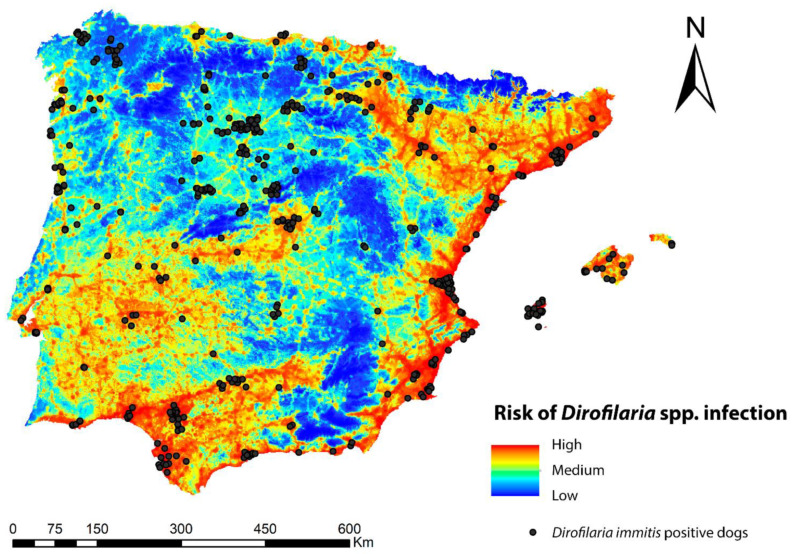
Ecological niche model of the risk of *Dirofilaria* spp. infection in the Iberian Peninsula and the Balearic Islands with the locations of infected dogs according to Alho et al. [77] and Montoya-Alonso et al. [13].

The regression calculation provided a significant positive relationship between the infection risk model and the prevalence of infected dogs per province (β ± SE = 3.32 ± 1.43 *p* < 0.05) (Figure 5). The risk model correctly classified the majority (>70%) of the existing records of *D. immitis*-infected dogs with very high to medium-high values, while less than 30% were misassigned to low or very low values (Figure 6).

### 3.5. Forward Projection of Culex pipiens

**Figure 7:**
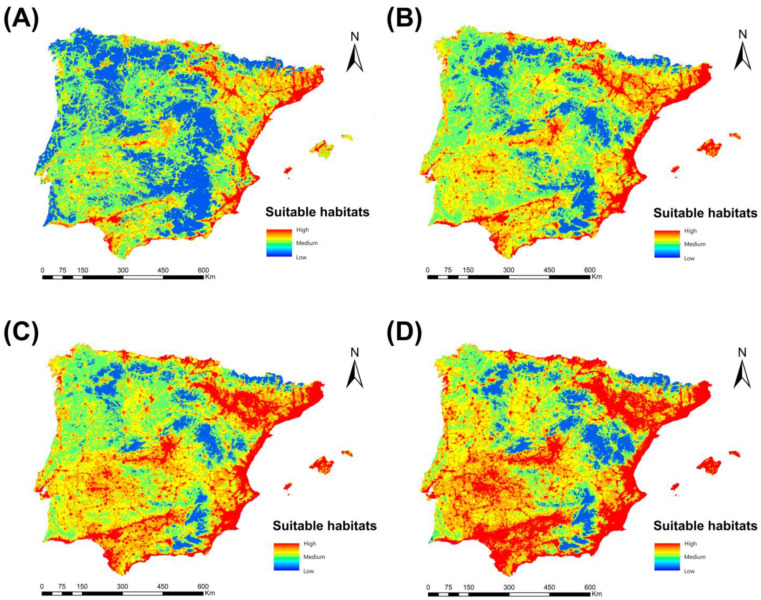
Suitable habitats for *Cx. pipens* at present (**A**) and their projections into the future, 2040 (**B**), 2060 (**C**), and 2080 (**D**), for the Iberian Peninsula and Balearic Islands under the climate change scenario RCP 8.5.

Regarding the change in range analysis, the projections for the future time periods 2040, 2060, and 2080, according to the climate change scenario RCP 8.5, showed a notable increase in the extension of the areas suitable for the presence of *Cx. pipiens* (Figure 7). Thus, the percentage gain of territory for *Cx. pipiens* in 2040 was 22.87%, rising to 44.53% in 2060 and increasing further in 2080 to 49.98%. From the point of view of the spatial distribution of suitable areas for this mosquito, by 2040, they will have increased in general throughout the peninsular territory, except in mountainous areas, which are still not very favorable. In 2060, these areas expand, extending outside the sphere of influence of the river basins and occupying intensely anthropized inland regions with abundant irrigated crops. Finally, in 2080, the favorable zones come to occupy practically the entire study territory, which is particularly evident in the central south, as well as in the northwest and southeast of the peninsula.

## 4. Discussion

This study provides a quantitative proposal of the risk of *Dirofilaria* spp. infection for the whole Iberian Peninsula (Spain and Portugal) and the Balearic Islands using the potential distribution of suitable habitats for *Cx. pipiens* and considering numerous predictor variables. Several species are responsible for the transmission of dirofilariosis in Europe [3,5]. However, in the Iberian Peninsula, only *Cx. pipiens* has been described as the only species transmitting *D. immitis* [28,29,30]. This fact, as well as the fact that it is the most abundant species in the study area [2,5], are the main factors that have led to the study of its potential distribution of suitable habitats.

Several predictive models of *Dirofilaria* spp. infection risk have been published in Europe, developed using GIS and based only on temperature records, to determine the number of generations of *Dirofilaria* spp. that can develop in the vector and, in some of them, the duration of annual disease transmission was also taken into account. In all these studies, an oceanic climate was assumed for the whole of Western Europe with sufficient humidity for the development of the vectors, so that regions of greater aridity were not taken into account [10,55,56,57,58,59,60,61,62,63]. For the Spanish part of the Iberian Peninsula, which includes the Balearic Islands, there is a previous methodologically simpler work, where the number of parasite generations and environmental and soil humidity were considered as environmental variables [7].

Our results indicated that for the Iberian Peninsula and the Balearic Islands as a whole, there was a possibility of infection risk. We found a robust and positive relationship between the risk of dirofilariosis and the observed prevalence of infested dogs in the study area, so that most of the positives (geo-referenced records of dogs infected by *D. immitis*) in the Iberian Peninsula and the Balearic Islands were located in areas of high/medium risk of infection.

It is known that bioclimatic conditions, mainly humidity and temperature, have a great impact on the extent and seasonality of dirofilariosis, largely due to the environmental requirements of its vectors [5]. In the case of the Iberian Peninsula and the Balearic Islands, there is a possibility of extrinsic development of *Dirofilaria* spp. larvae in vectors in most of their territory, with low rainfall being a limiting factor for the establishment of mosquito populations. This circumstance, however, can be locally compensated by the presence of water bodies (natural or artificial), irrigated crops, or cities that offer an ideal habitat for mosquito breeding and where high prevalences of canine dirofilariosis have sometimes been obtained [64,81,82,83,84,85]. Precisely, and as indicated in the results, the variables of the human footprint and irrigation density had the greatest statistical weight in our *Cx. pipiens* ENM, which means that the areas with the highest *Cx. pipiens* presence corresponded to those with a high population density and a high concentration of canals and irrigated areas (eastern and southern coastal strip of the Iberian Peninsula, as well as the surroundings of the basins of the large rivers). In addition, the wild reservoirs infected by *D. immitis* (wolf, fox, and Iberian lynx, among others) in the Iberian Peninsula [5], which lack parasitic control, are another factor to take into account regarding the risk of infection, in order to emphasize our model. However, we do not have data on their geolocation, which could limit the results of our model.

The result of our future projections under climate change scenarios revealed a displacement of the current range of *Cx. pipiens* into new territories. By 2040, the percentage gain of territory of this vector in the peninsula will be no less than 27.87%, and the gains will be 44.53% and 49.98% for the projections of 2060 and 2080, respectively. This will mean a strong increase in its potential area, mainly toward the northwest of the peninsula, resulting in an increased risk of infection with *Dirofilaria* spp. in the same areas. This circumstance coincides with that reported by Hongoh et al. [86], which confirms the latitudinal displacement of the distribution area of *Cx. pipiens*, and therefore, it is possible that this change will influence the range and transmission dynamics of certain diseases transmitted by this species, as predicted by our study. According to these results, and in line with the predictions of other studies [47,50,61], the risk areas for *Dirofilaria* transmission, as for other vector-borne diseases, will change spatially, moving dangerously close to areas where they were previously absent. The effect of climate change on the incidence of these diseases seems to become more pronounced at the extremes of the temperature ranges in which transmission of the disease occurs [87], and consequently, there will be major changes in its seasonality and distribution in the climatic margins of cold and temperate zones [88], coinciding with what has been observed in our territory.

## 5. Conclusions

Successful predictive modeling of vector-borne diseases requires the incorporation of predictor variables that influence the environmental dynamics of vector-borne diseases, which in turn implies considering the dynamics of the species that act as their vectors, allowing the creation of much more spatially accurate risk models. From our results, the incorporating of the suitable habitats for *Cx. pipiens* and the potential number of *Dirofilaria* spp. generations as critically important variables in the risk model represents a novel contribution to substantially improve the predictive power of existing studies. The risk of infection is high in the eastern coastal strip and the south of the Iberian Peninsula and the Balearic Islands, as well as in the areas surrounding the basins of the main rivers, and the lowest risk is located in the higher-altitude areas. In addition, future projections under climate change scenarios allowed us to visualize a potential increase in the distribution of *Cx. pipiens* in the Iberian Peninsula, which will increase the potential risk of *Dirofilaria* spp. infection.

This model will help veterinary and public health professionals to perform more efficient and localized prevention and control tasks in relation to dirofilariasis, taking into account the specific situation of each population. Further studies are needed to further assess the risk of infection at the local level in order to take precautions to prevent the possible spread of the disease in the coming years.

## Figures and Tables

**Table 1:** Analysis of the contribution of the 13 environmental and bioclimatic variables to the ecological niche model for *Cx. pipiens*.

Variable	Percent Contribution
Human footprint	56.7%
BIO_8_ (mean temperature of the wettest quarter)	27.2%
BIO_4_ (temperature seasonality)	3.6%
Rivers	3.3%
Herbaceous density	2.1%
BIO_9_ (mean temperature of the driest quarter)	1.9%
BIO_15_ (precipitation seasonality)	1.5%
BIO_1_ (annual mean temperature)	1.2%
Shrub density	0.7%
Water bodies	0.6%
Irrigated crops	0.6%
BIO_3_ (isothermality)	0.4%
BIO_12_ (annual precipitation)	0.2%

## Data Availability

Not applicable.

## Supplementary Materials

The following supporting information can be downloaded at https://www.mdpi.com/article/10.3390/ani13111764/s1: Supplementary Figure S1: Visual representation of BIO1 (annual mean temperature) in the Iberian Peninsula and Balearic Islands. Supplementary Figure S2: Visual representation of BIO3 (isothermality) in the Iberian Peninsula and Balearic Islands. Supplementary Figure S3: Visual representation of BIO_4_ (temperature seasonality) in the Iberian Peninsula and Balearic Islands. Supplementary Figure S4: Visual representation of BIO_8_ (mean temperature of the wettest quarter) in the Iberian Peninsula and Balearic Islands. Supplementary Figure S5: Visual representation of BIO_9_ (mean temperature of the driest quarter) in the Iberian Peninsula and Balearic Islands. Supplementary Figure S6: Visual representation of BIO_12_ (annual precipitation) in the Iberian Peninsula and Balearic Islands. Supplementary Figure S7: Visual representation of BIO_15_ (precipitation seasonality) in the Iberian Peninsula and Balearic Islands. Supplementary Figure S8: Visual representation of the human footprint in the Iberian Peninsula and Balearic Islands. Supplementary Figure S9: Visual representation of irrigated crops in the Iberian Peninsula and Balearic Islands. Supplementary Figure S10: Visual representation of rivers in the Iberian Peninsula and Balearic Islands. Supplementary Figure S11: Visual representation of bodies of water in the Iberian Peninsula and Balearic Islands. Supplementary Figure S12: Visual representation of herbaceous density in the Iberian Peninsula and Balearic Islands. Supplementary Figure S13: Visual representation of shrub density in the Iberian Peninsula and Balearic Islands.
