# Supplementary figures and images for: Current Risk of Dirofilariosis Transmission in the Iberian Peninsula (Spain and Portugal) and the Balearic Islands (Spain) and Its Future Projection under Climate Change Scenarios
Source: Animals (Basel). 2023 May 26;13(11):1764. doi: 10.3390/ani13111764 (PMC10251949; doi:10.3390/ani13111764)

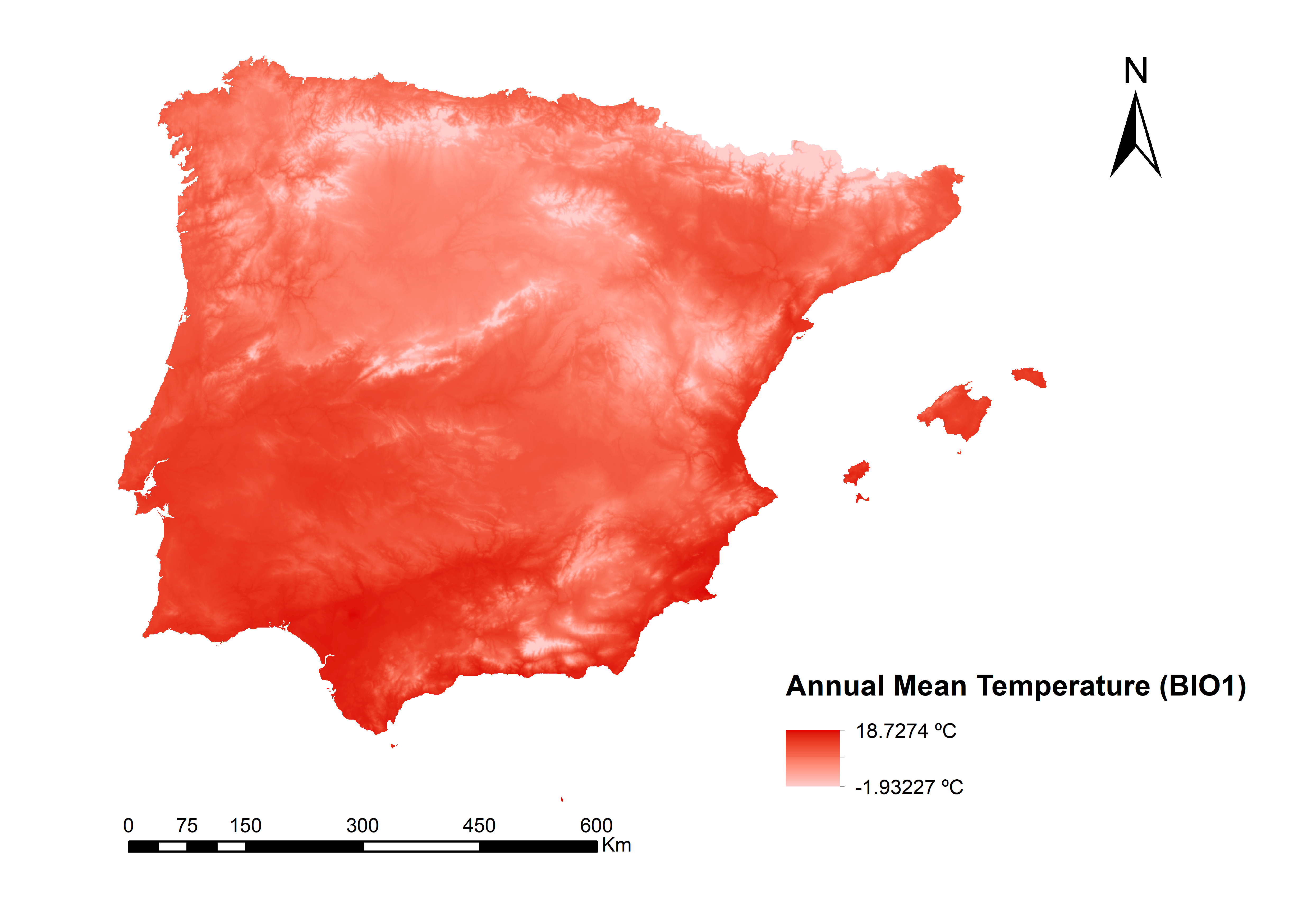

Supplement: Supplementary file 1 [file animals-13-01764-s001.zip › Sup S1. BIO1.tif]

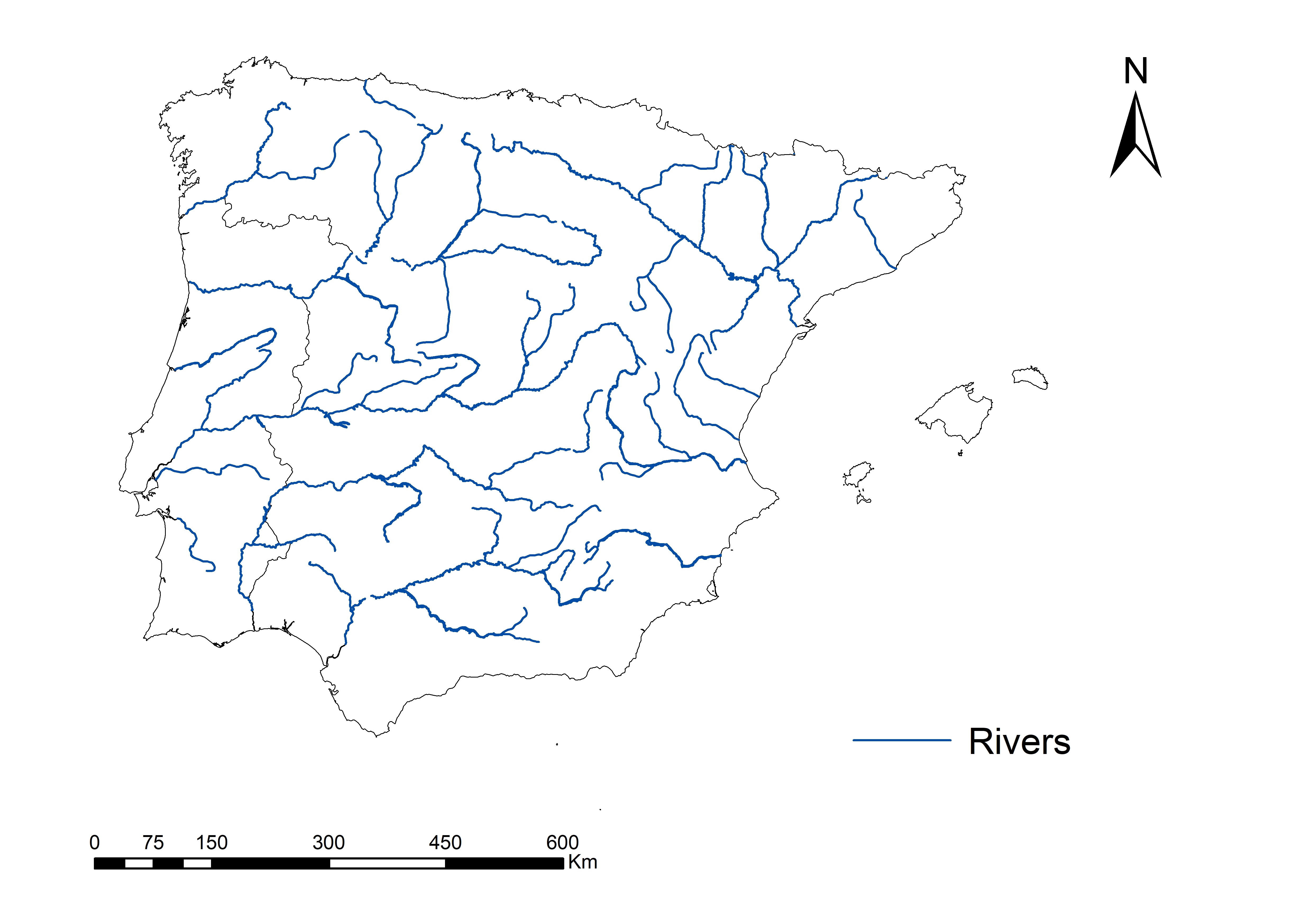

Supplement: Supplementary file 1 [file animals-13-01764-s001.zip › Sup S10. Rivers.tif]

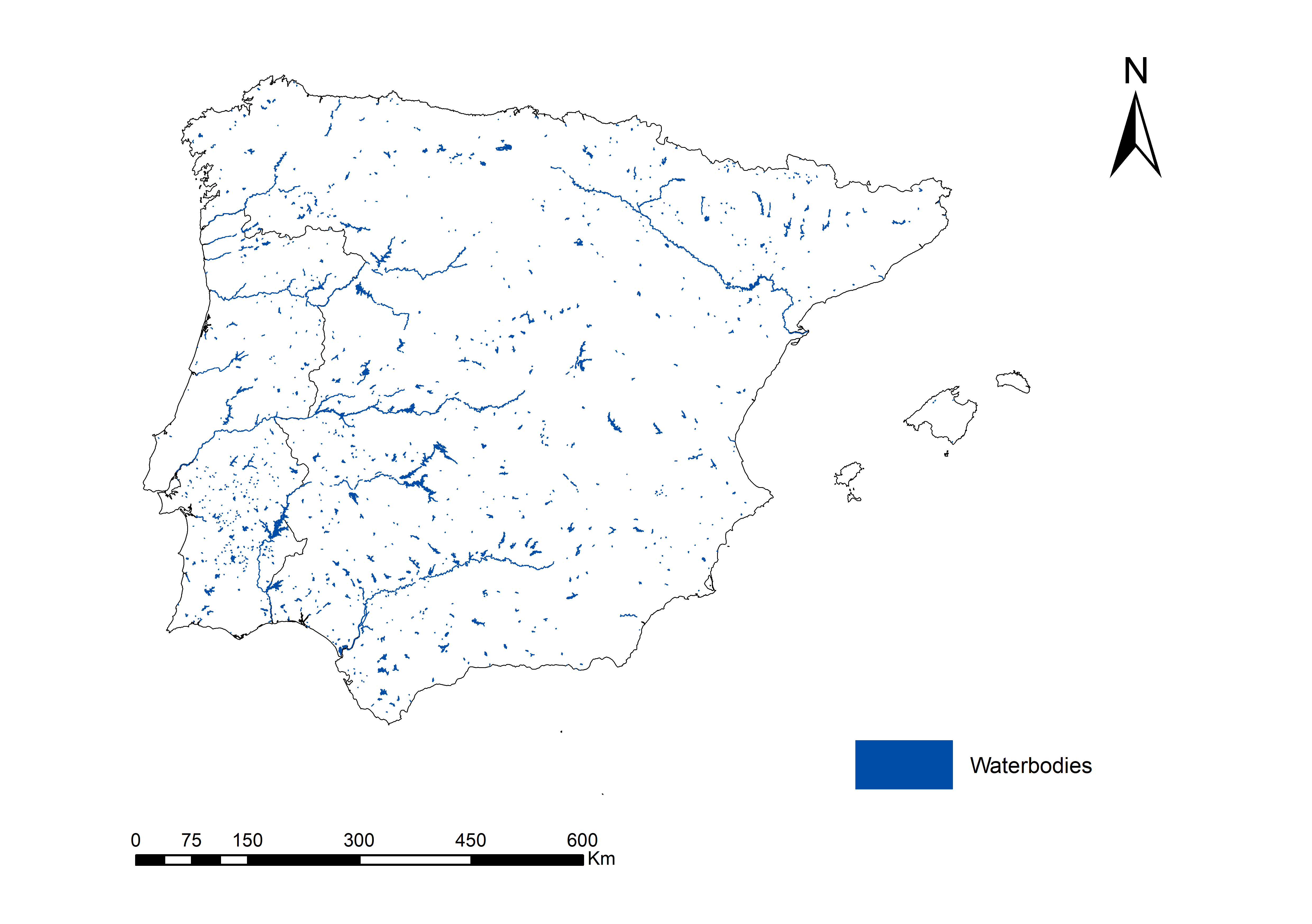

Supplement: Supplementary file 1 [file animals-13-01764-s001.zip › Sup S11. Water Bodies.tif]

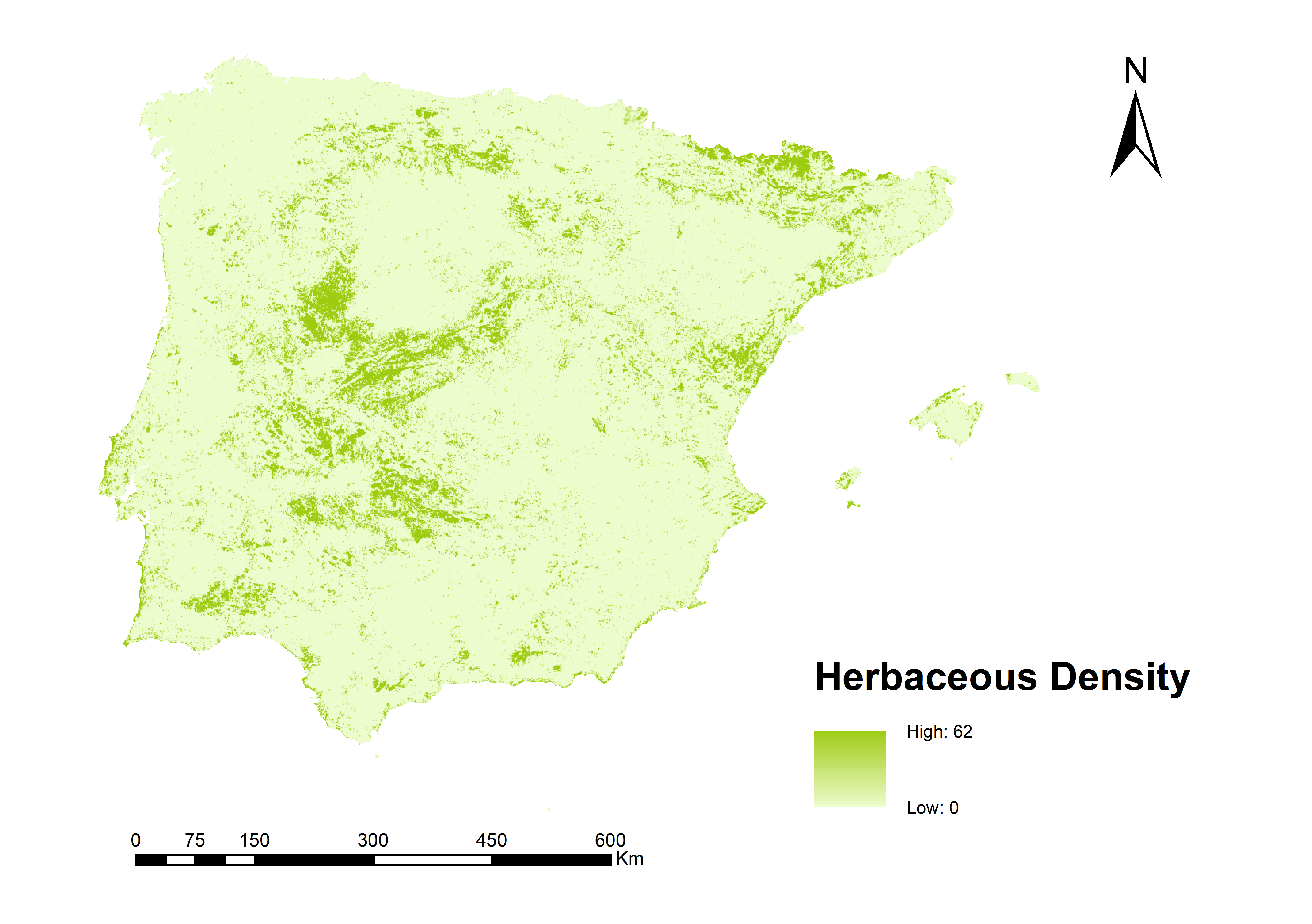

Supplement: Supplementary file 1 [file animals-13-01764-s001.zip › Sup S12. Herbaceous density.tif]

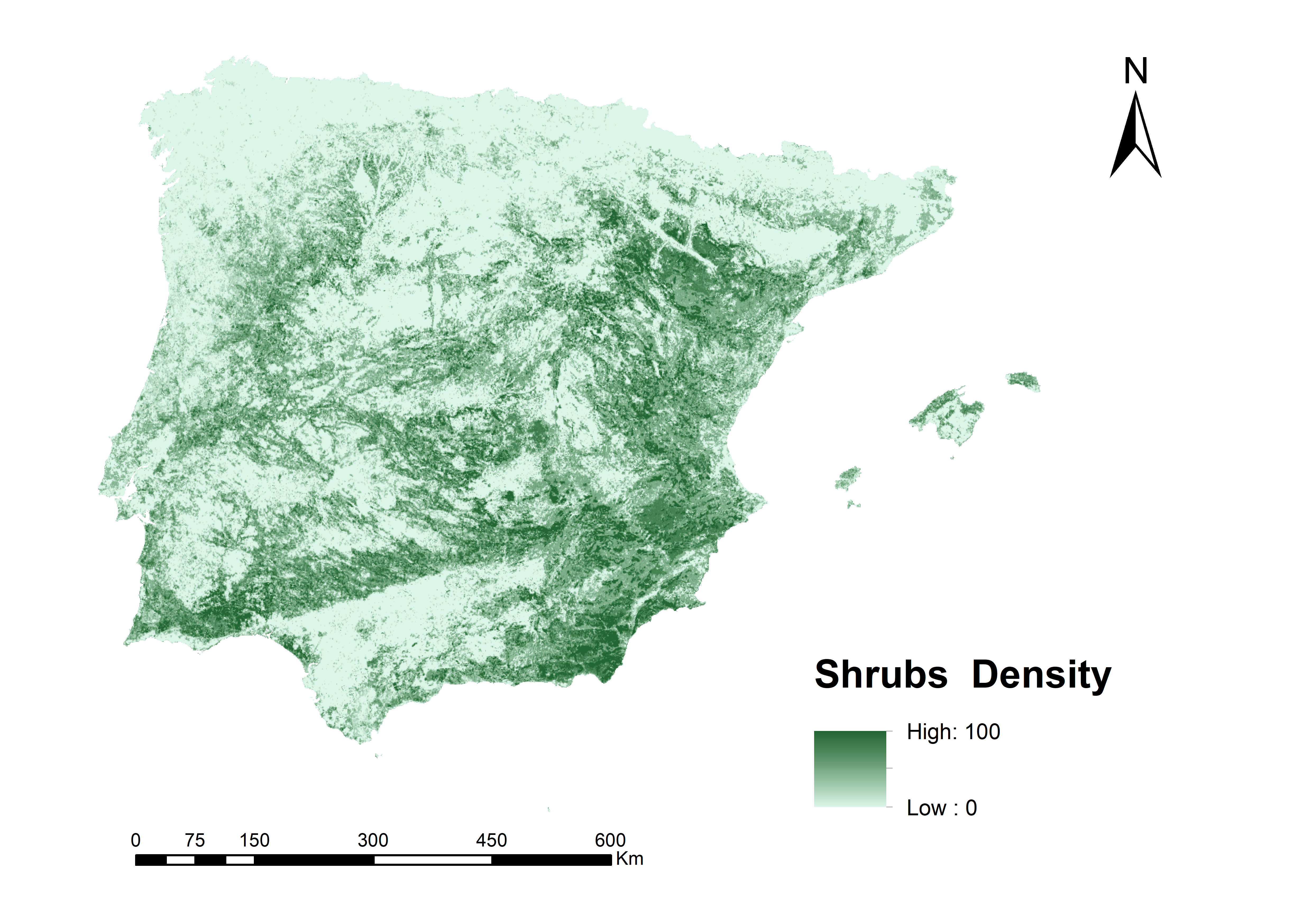

Supplement: Supplementary file 1 [file animals-13-01764-s001.zip › Sup S13. Shrubs density.tif]

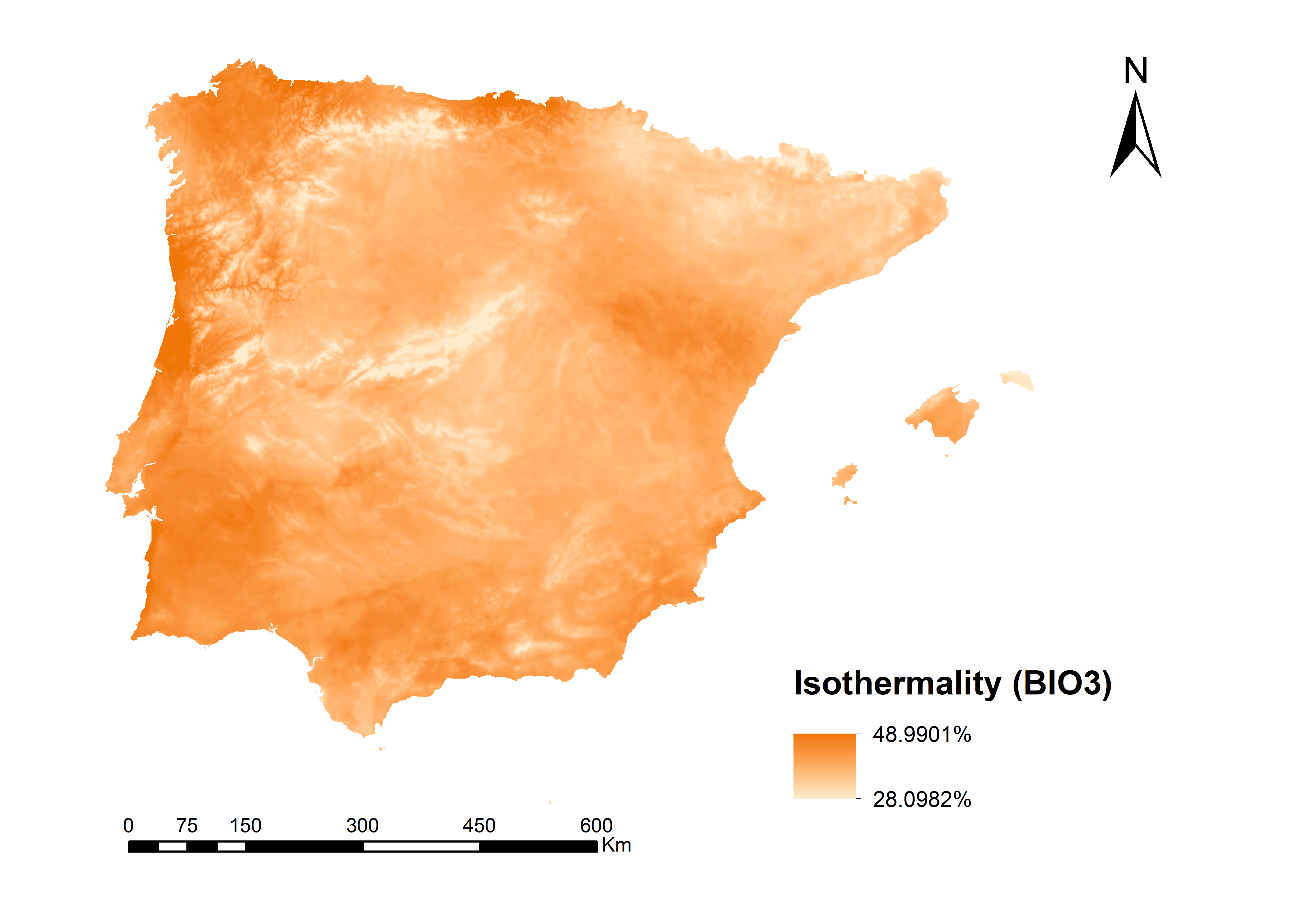

Supplement: Supplementary file 1 [file animals-13-01764-s001.zip › Sup S2. BIO3.tif]

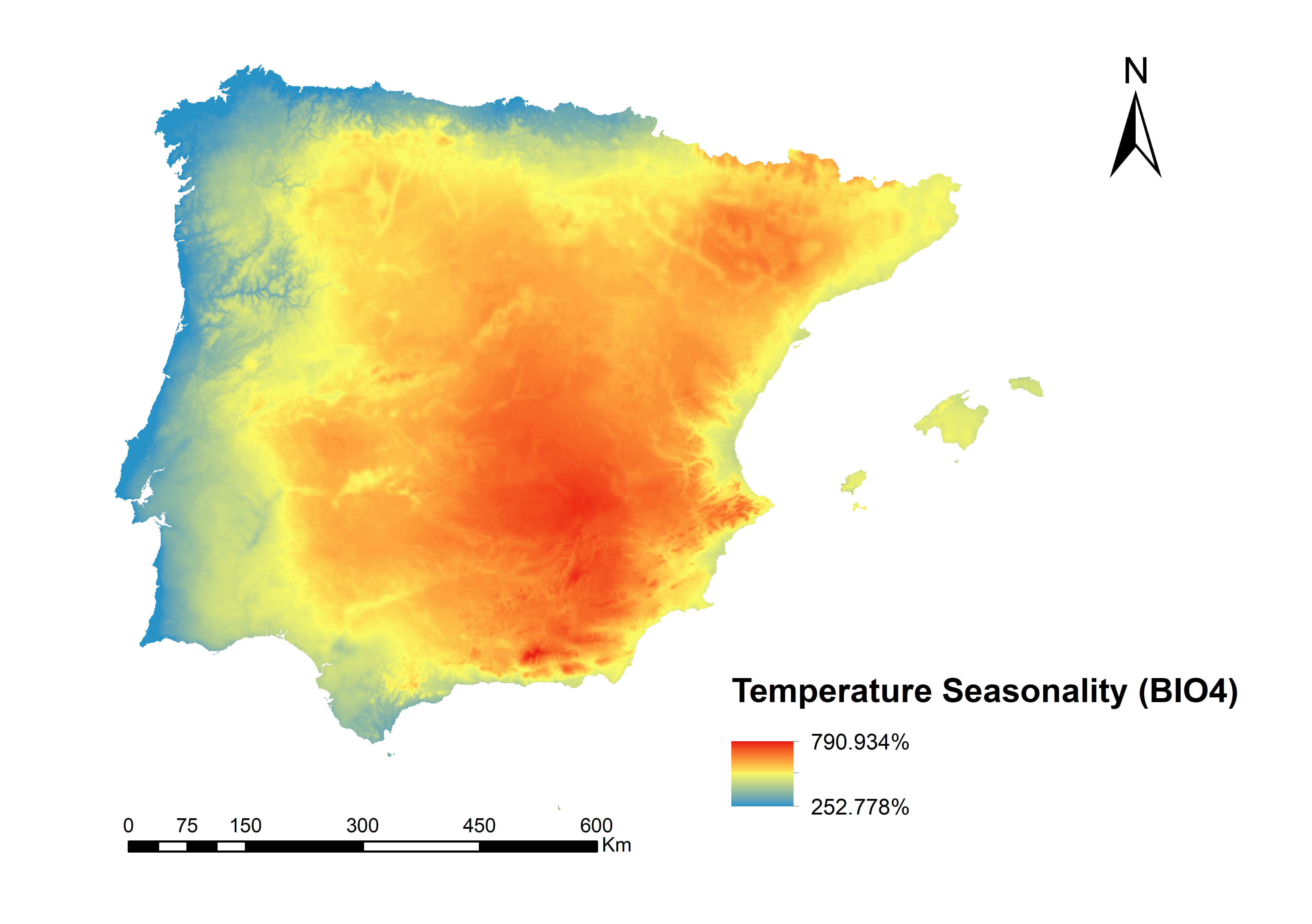

Supplement: Supplementary file 1 [file animals-13-01764-s001.zip › Sup S3. BIO4.tif]

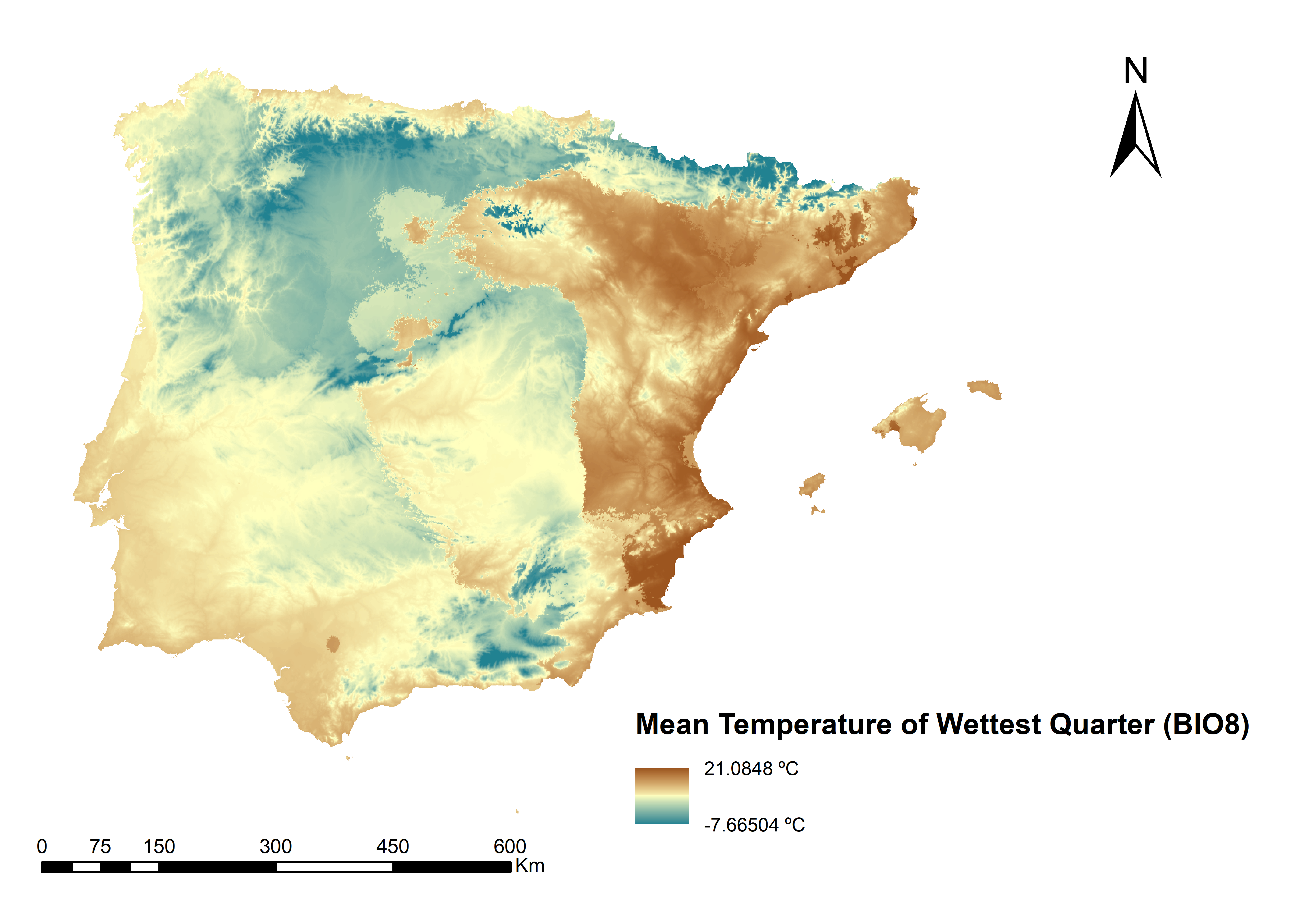

Supplement: Supplementary file 1 [file animals-13-01764-s001.zip › Sup S4. BIO8.tif]

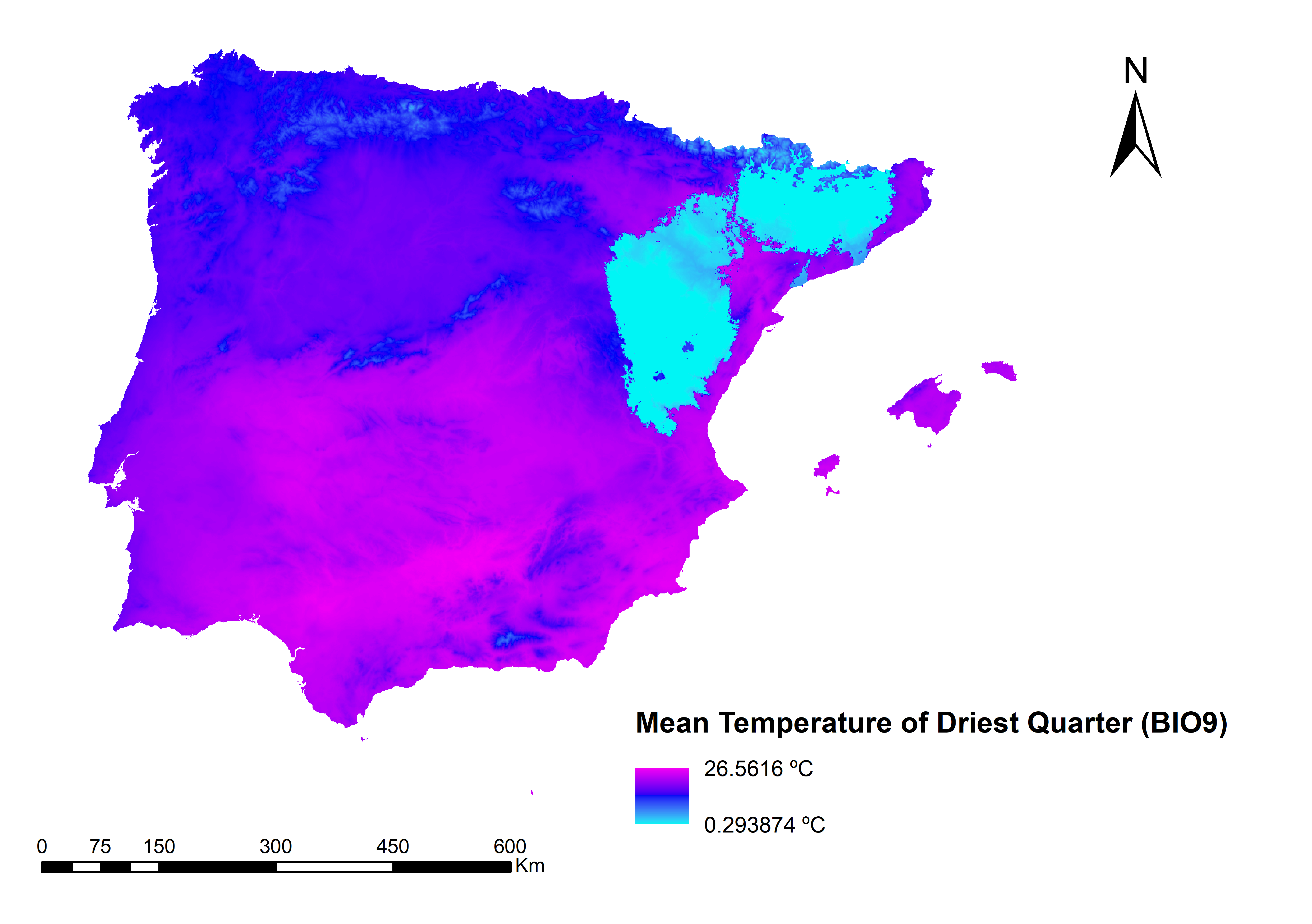

Supplement: Supplementary file 1 [file animals-13-01764-s001.zip › Sup S5. BIO9.tif]

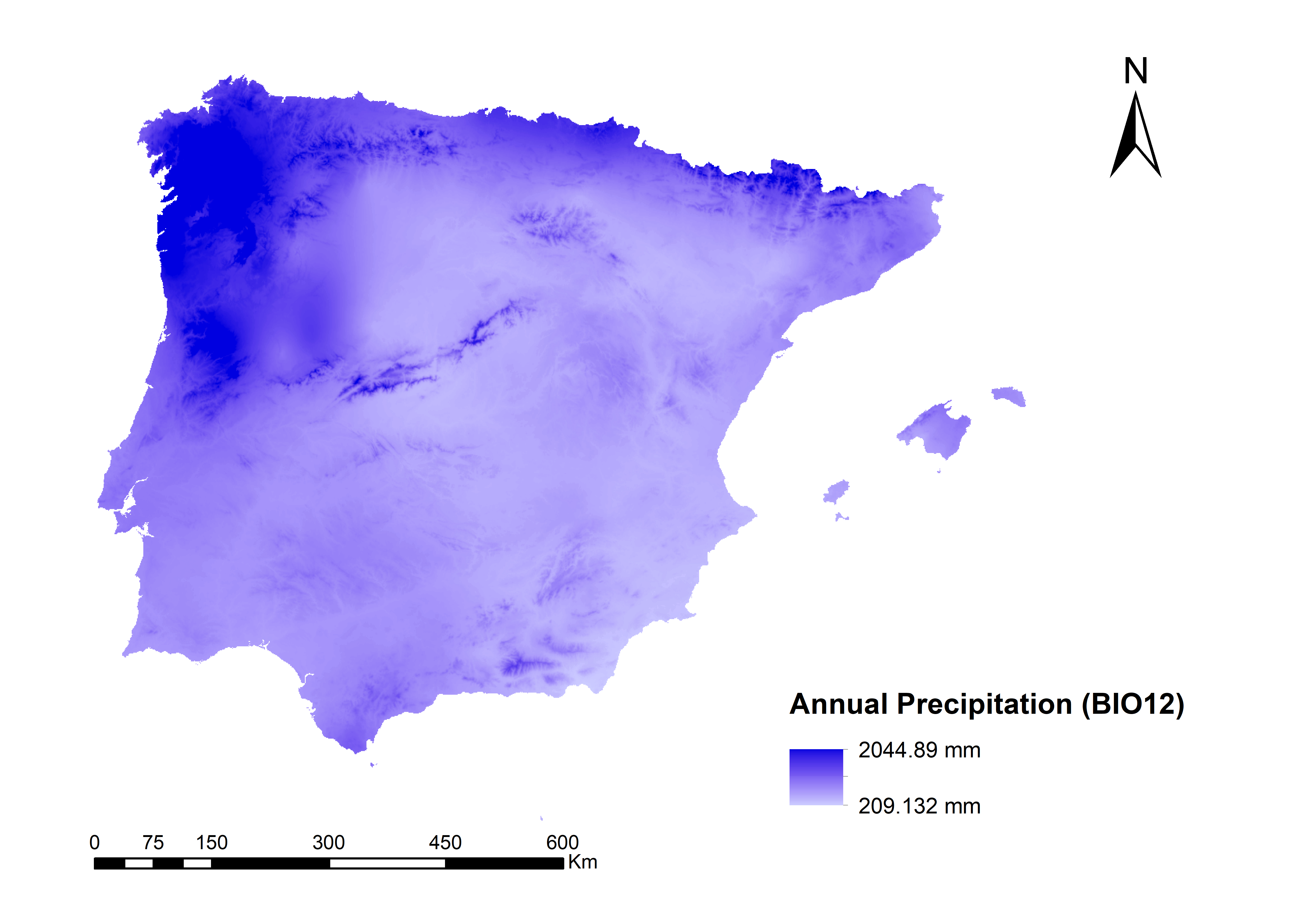

Supplement: Supplementary file 1 [file animals-13-01764-s001.zip › Sup S6. BIO12.tif]

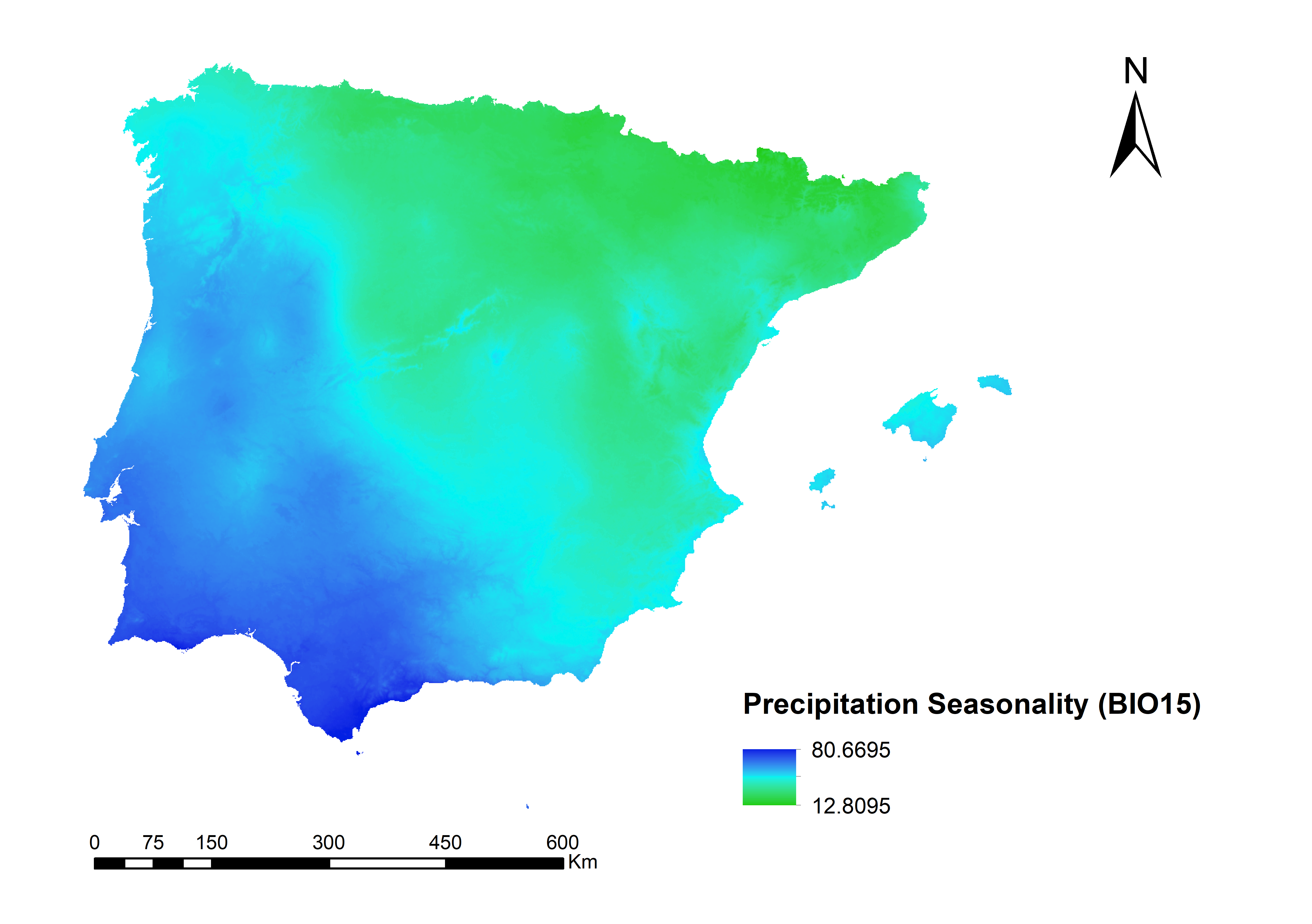

Supplement: Supplementary file 1 [file animals-13-01764-s001.zip › Sup S7. BIO15.tif]

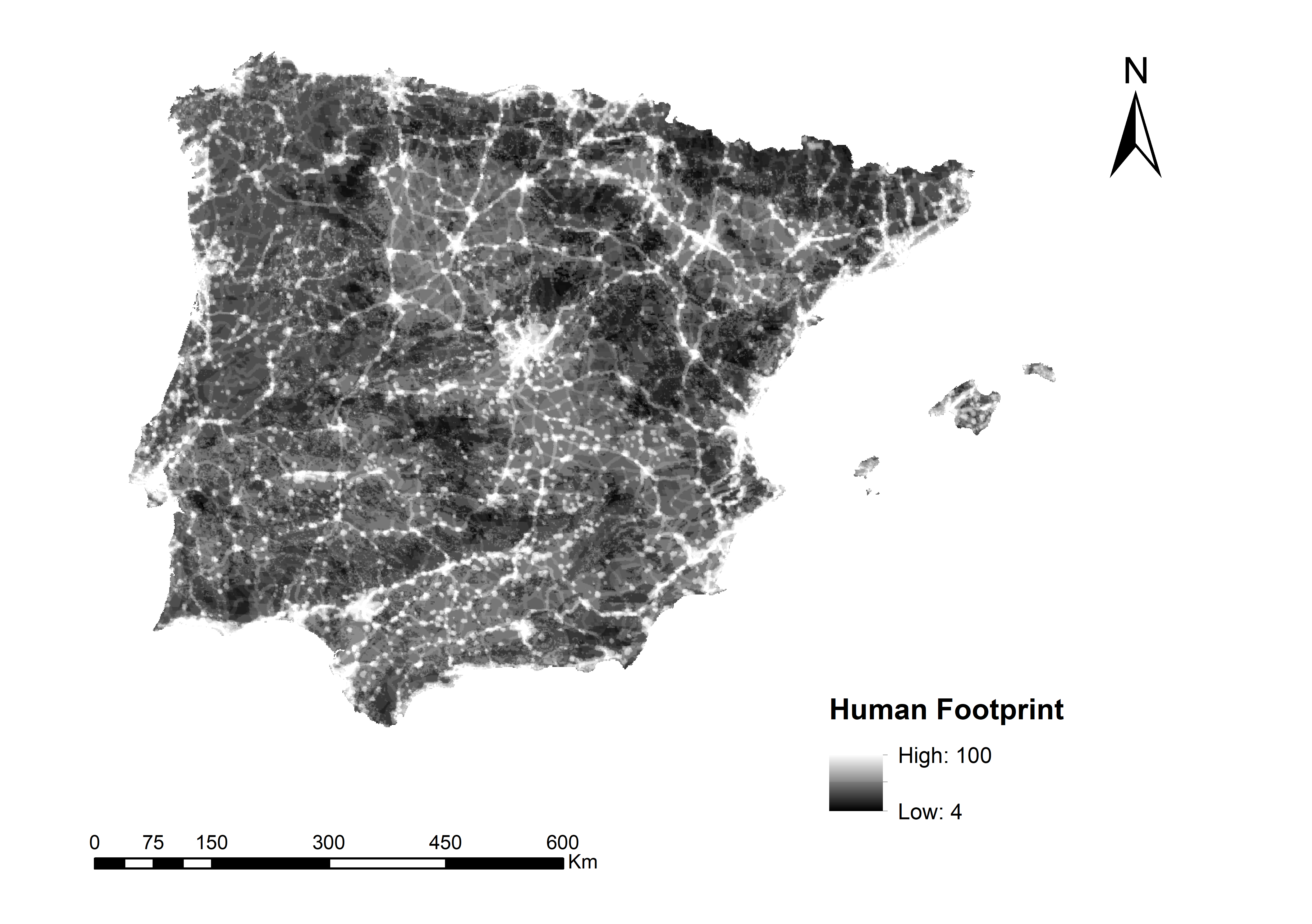

Supplement: Supplementary file 1 [file animals-13-01764-s001.zip › Sup S8. Human Footprint.tif]

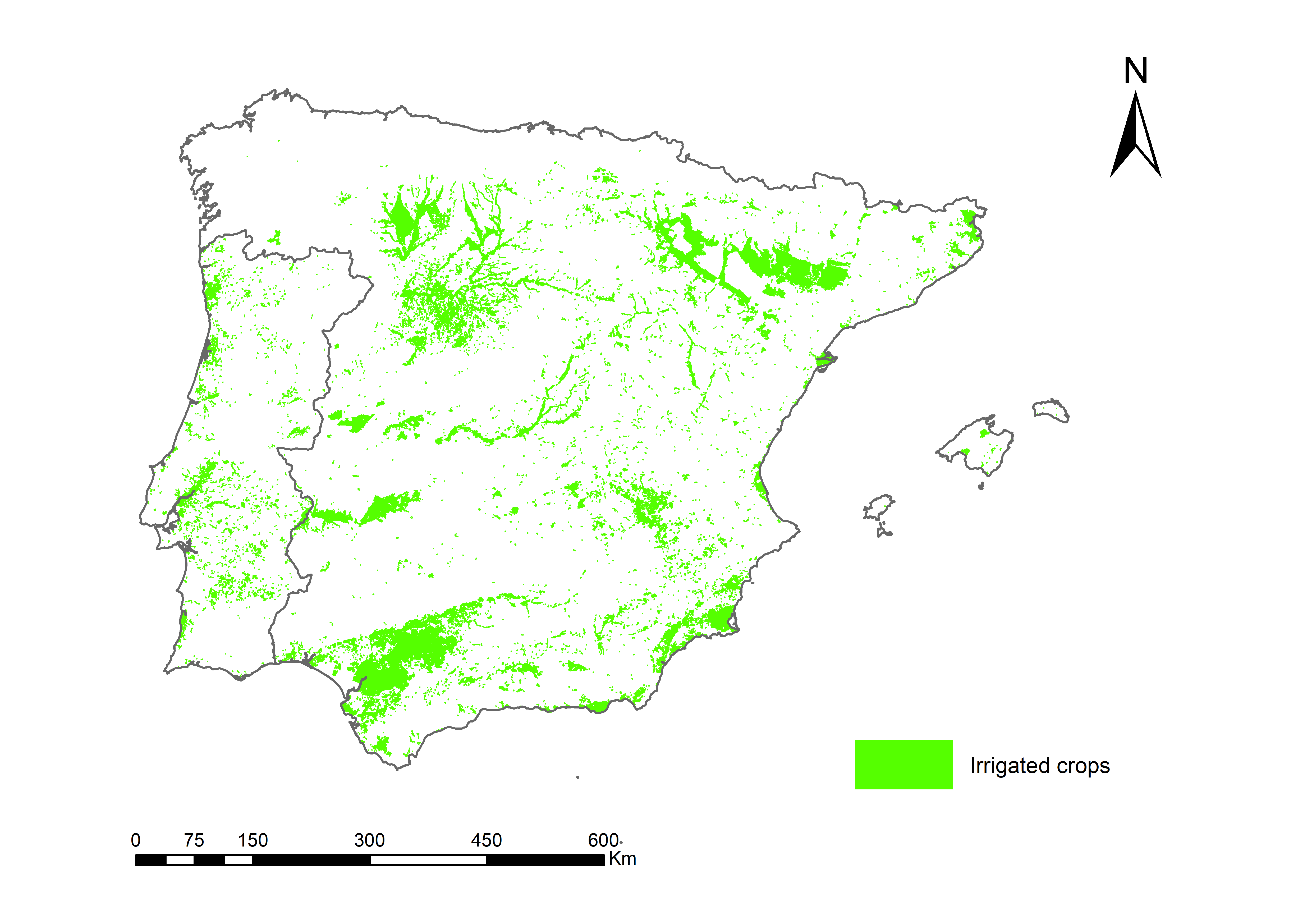

Supplement: Supplementary file 1 [file animals-13-01764-s001.zip › Sup S9. Irrigated crops.tif]
